# Primary Health Care in Nigeria: 24 Years after Olikoye Ransome-Kuti’s Leadership

**DOI:** 10.3389/fpubh.2017.00048

**Published:** 2017-03-13

**Authors:** Bolaji Samson Aregbeshola, Samina Mohsin Khan

**Affiliations:** ^1^Community Health and Primary Care, University of Lagos, Lagos, Nigeria; ^2^Public Health Sciences, Karolinska Institutet, Stockholm, Sweden

**Keywords:** primary health care, Nigeria, Olikoye Ransome-Kuti, health systems, Alma Ata Declaration

Policy makers need to strengthen and revitalize primary health care (PHC) in Nigeria. This opinion aims to inform policy decisions and actions by examining the evolution of PHC in Nigeria, the role of Professor Olikoye Ransome-Kuti in the implementation of Alma Ata Declaration, the present state of PHC, the challenges and opportunities in implementing PHC in Nigeria, as well as ways to maximize the opportunities.

In 1960, there was no strong focus on health systems development. Policy makers and political actors made efforts to establish and expand health-care infrastructures with more emphasis placed on curative medicine rather than preventive medicine (1). From 1975 to 1980, health system development was initiated with PHC as the cornerstone (1). The National Basic Health Services Scheme (NBHSS) was developed based on a PHC approach (1). Unfortunately, the NBHSS program could not achieve its goals due to implementation challenges; hence, PHC services were not delivered across Nigeria (2). In 1985, Professor Olikoye Ransome-Kuti was appointed the Minister of Health. Professor Ransome-Kuti adopted PHC in 52 local government areas as models based on Alma Ata Declaration of 1978 (3). Furthermore, Nigeria’s first comprehensive national health policy based on PHC was launched in 1988 (3). From 1986 to 1990, Professor Olikoye Ransome-Kuti expanded PHC to all local governments, achieved universal child immunization of over 80%, and devolved responsibility for PHC to local government areas (3). Professor Olikoye Ransome-Kuti worked assiduously between 1985 and 1992 to implement PHC policy based on the Alma Ata Declaration for the benefit of the Nigerian population. Professor Olikoye Ransome-Kuti introduced a comprehensive national health policy with a focus on PHC, placed emphasis on preventive medicine and health-care services at the grass root, ensured exclusive breast feeding practice, introduced free immunization to children, encouraged the use of oral rehydration therapy by nursing mothers, made compulsory the recording of maternal deaths, and encouraged continuous nationwide vaccination and pioneered effective HIV/AIDS campaign. In 1992, the National Primary Health Care Development Agency (NPHCDA) was established to ensure that the PHC agenda is continued and sustained (1, 3). The military takeover of government that occurred in 1993 brought to an end the giant strides recorded under the leadership of Professor Olikoye Ransome-Kuti from 1985 to 1992.

Twenty-four years after the leadership of Professor Olikoye Ransome-Kuti, the need to strengthen the PHC in Nigeria is relevant as ever before. The current state of PHC system in Nigeria is appalling with only about 20% of the 30,000 PHC facilities across Nigeria working (4). Presently, most of the PHC facilities in Nigeria lack the capacity to provide essential health-care services, in addition to having issues such as poor staffing, inadequate equipment, poor distribution of health workers, poor quality of health-care services, poor condition of infrastructure, and lack of essential drug supply (5). In part, problems with the implementation of PHC in Nigeria are related to the hand over in 1980s to the local government administration, which is the weakest level of government (6, 7). The impact of local government administration on the people in Nigeria still remains a subject of intense debate and argument (8). Conversely, the Alma Ata Declaration has been successfully implemented by countries such as Thailand, Cuba, China, and Mexico (9).

The PHC under one roof (PHCUOR) policy was formulated in 2011 to address the problem of fragmentation in PHC and ensure the integration of PHC services under one authority. Its impact is yet to be felt on health status and utilization of PHC in Nigeria since PHC under one roof became a national policy only few years ago. The inability of PHC centers to provide basic medical services to the Nigerian population have made both secondary and tertiary health-care facilities experience an influx of patients. This has had its toll on the secondary and tertiary levels of care (10).

Part of the Alma Ata Declaration is that health is a fundamental human right and governments should be responsible for the health of the people (11). Health is rarely seen as a fundamental human right by policy makers in Nigeria; hence, the inability to implement the Abuja Declaration in which African heads of state pledge to set a target of earmarking at least 15% of their annual budget to improve the health sector. Increasing investment in health of the people has been a challenge for decision makers in spite of evidence showing the link between health and economic development (12). Many African countries, including Nigeria, fall short of the Abuja Declaration of 2001 in spite of the pledge by heads of state of African Union countries (13). It is established that the successful implementation of PHC in any country requires adequate financial resources (14).

The Alma Ata Declaration also encourages partnership and cooperation with other related sectors of the economy in the development and implementation of PHC (11). Unfortunately, related sectors are also battling a series of challenges and are yet to deliver on their responsibilities to the people. A return to Alma Ata principle would guide further development.

The establishment of NPHCDA and the 30,000 PHC facilities across Nigeria provide an opportunity for the effective implementation of PHC in Nigeria. Therefore, governments have to maximize the opportunity provided by existing PHC facilities to make PHC sustainable in order to strengthen Nigeria’s health-care system. The running of PHC facilities would be more effective if federal and state governments took over their administration from the local governments.

In conclusion, PHC policy in Nigeria can be strengthened through the implementation of the Abuja Declaration, thereby increasing domestic resources for health and improved budgetary allocation for the management of PHC. Governments should redirect resources for health care from curative services to preventive services in order to improve PHC infrastructures, encourage the migration of health workers from urban areas to rural areas, and provide acceptable level of health-care services for all, thereby reducing the gross inequality in health status of the people. Political actors and policy makers could guarantee the right to health of all citizens by signing and implementing the necessary legislation.

## Author Contributions

The authors made substantial contribution to the conception and design of the manuscript, drafted the manuscript and revised it for important intellectual content, approved the final manuscript and this submission, and assumed responsibility for the manuscript.

## Conflict of Interest Statement

The authors declare that the research was conducted in the absence of any commercial or financial relationships that could be construed as a potential conflict of interest.
